# Supplementary material for: Combining simulation modeling and stable isotope analyses to reconstruct the last known movements of one of Nature’s giants
Source: PeerJ. 2019 Oct 18;7:e7912. doi: 10.7717/peerj.7912 (PMC6802580; doi:10.7717/peerj.7912)
Supplement: Supplemental Information 1 [file peerj-07-7912-s001.pdf]

# Supplemental Information from:
# *Reconstructing the last known movements of one of Nature's giants*

Clive N. Trueman, Andrew L. Jackson, Katharyn S. Chadwick,
Ellen J. Coombs, Sarah Magozzi, Richard C. Sabin
and Natalie Cooper

| | Behavioural state | | |
|---|---|---|---|
| **Parameters** | **Foraging** | **Migrating north** | **Migrating south** |
| Behavioural phase one (1095 days) | Jan - Dec | NA | NA |
| Behavioural phase two (1278 days) | June - Oct | April - May | Nov - March |
| Pregnancy, nursing, return (447 days) | Jan - Dec | NA | NA |
| Post nursing migration (100 days) | NA | Jan - June | NA |
| Minimum sea surface temperature (°C) | 3 | 3 | 3 |
| Maximum sea surface temperature (°C) | 25 | 25 | 25 |
| Maximum daily distance traveled (km)* | $N(\mu = 50, \sigma = 25)$ | $N(\mu = 150, \sigma = 75)$ | $N(\mu = 150, \sigma = 75)$ |
| Plankton biomass threshold for remaining to forage (units) | 50 | NA | NA |

Table S1: Model parameters for agent-based model of whale movement. See Figure S5 and text for more details. *During behavioural phase one, mean maximum daily distance traveled is set to 20km.

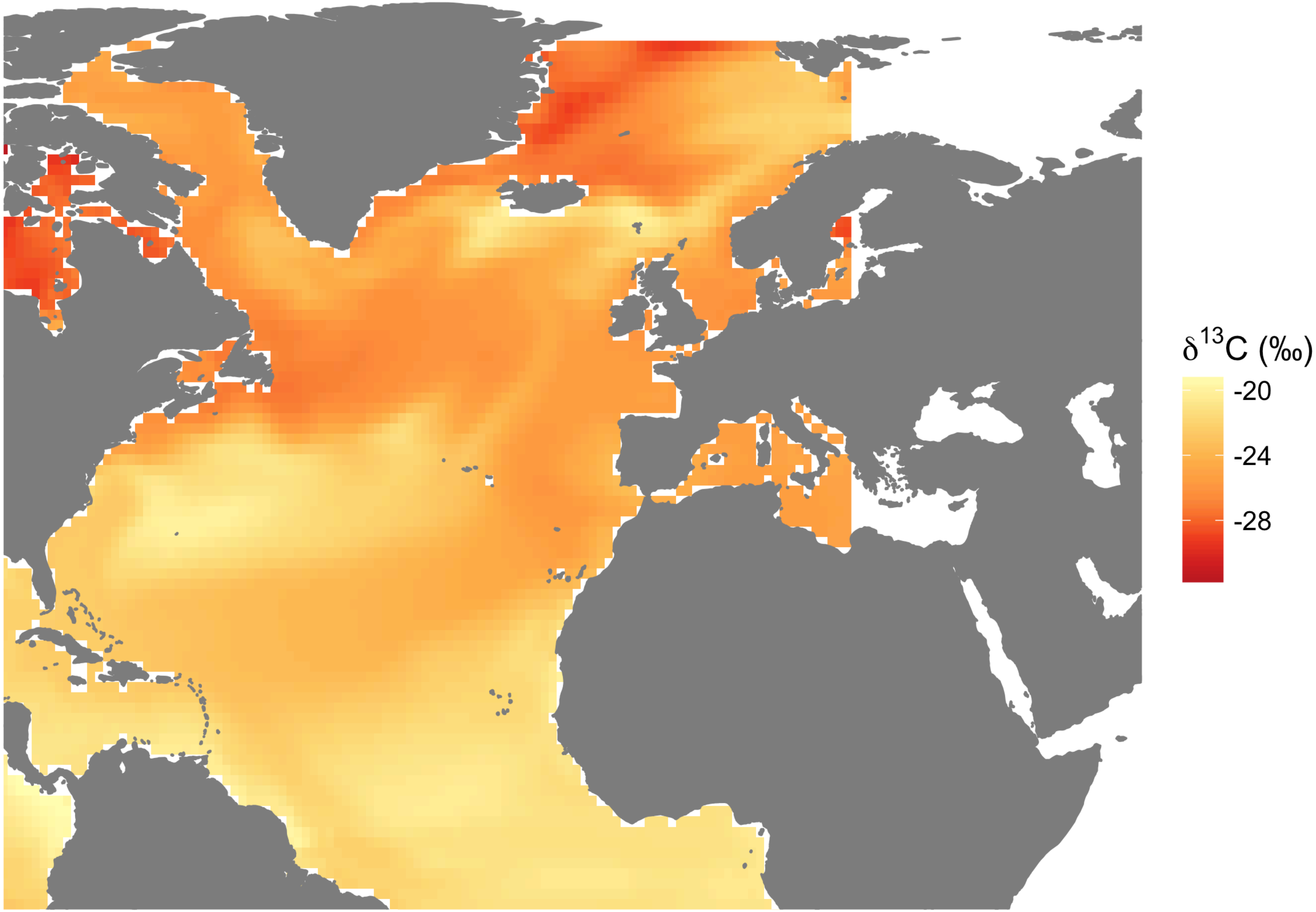


Figure S1: Spatial model (isoscape) of model-simulated biomass, using weighted annual average $\delta^{13}$C values from Magozzi *et al.* (2017).

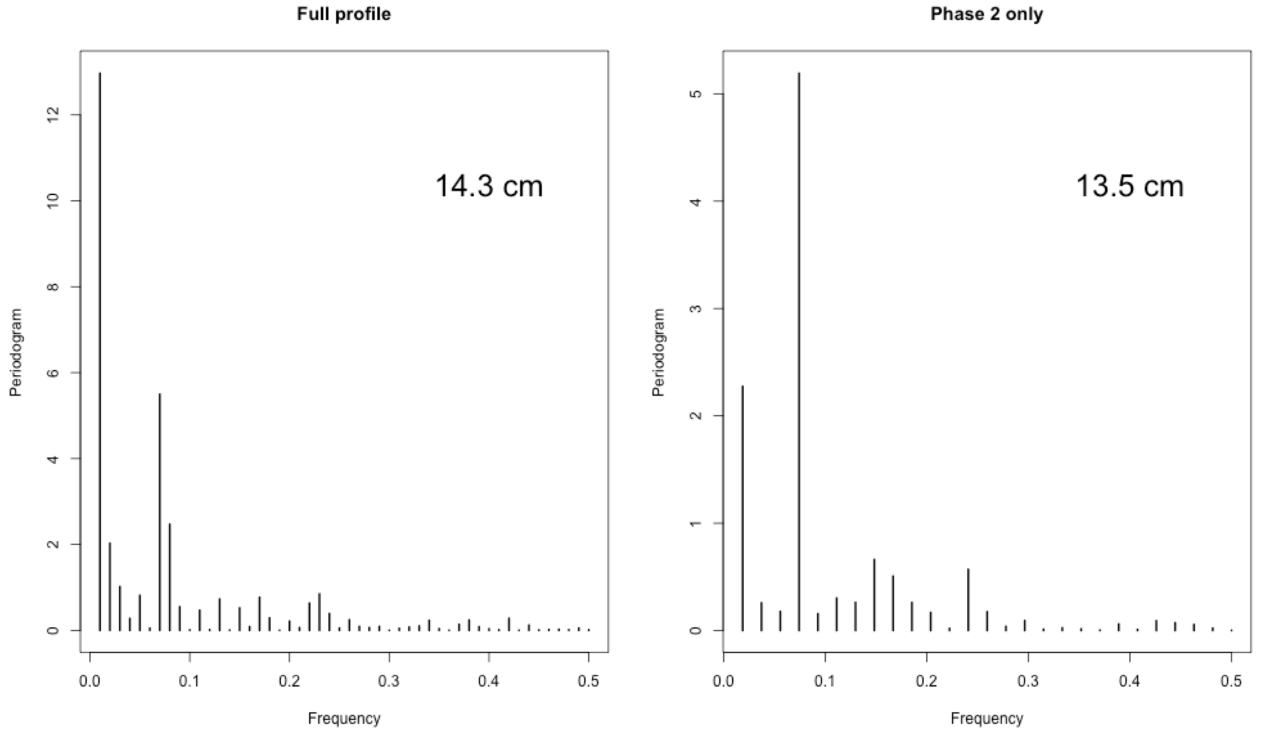


Figure S2: Fourier analysis of $\delta^{15}$N values of the full (A) and behavioural phase two only (B) components of the baleen plate. Inset values are the highest frequency spectral densities (in cm). Periodic fluctuation with a distance of 13.5cm along the baleen plate is consistently recovered in behavioural phase two where the whale engaged in latitudinal migrations. Note that we do not separately graph behavioural phases one or three here because they do not show clear periodicity.

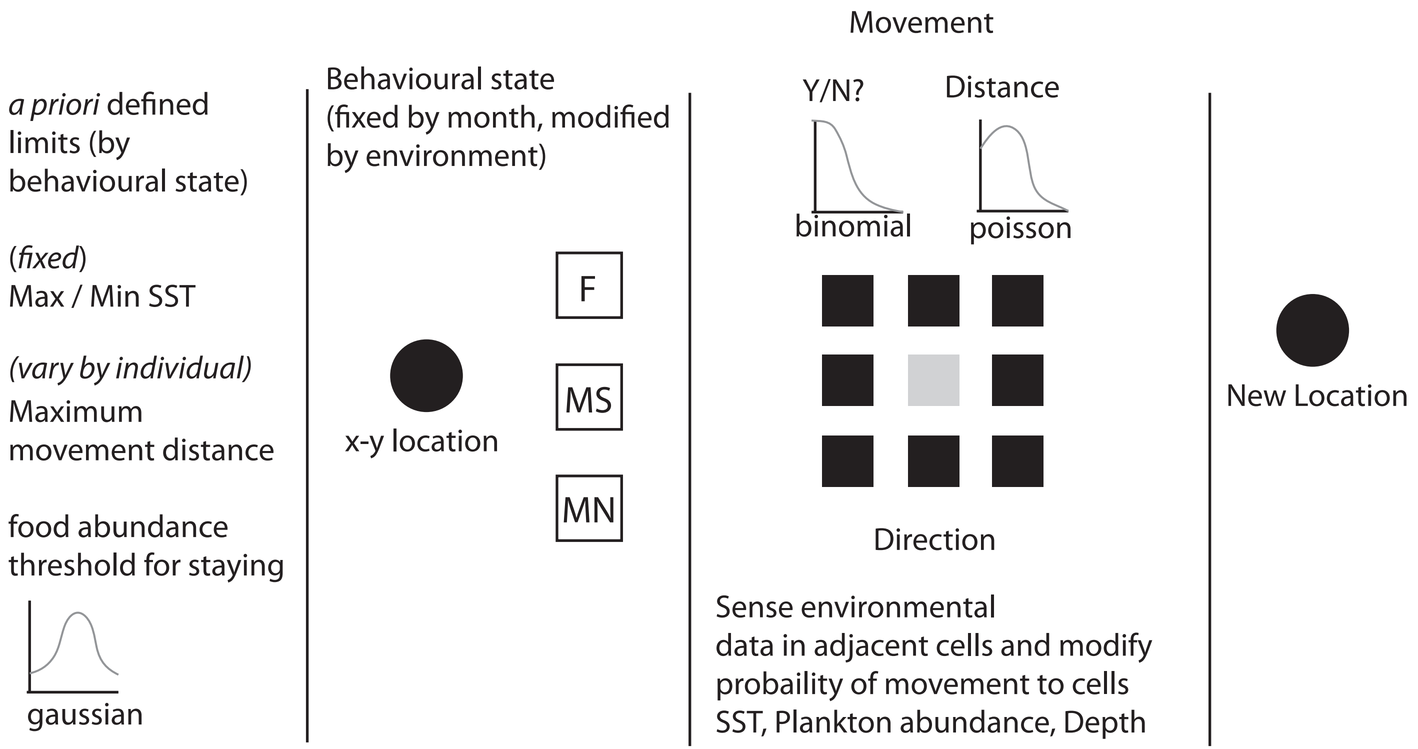


Figure S3: Schematic structure of the agent-based model used to generate hypothetical movement tracks. At the start of each simulation, max and min sea surface temperature (SST) and food availability thresholds are fixed for all individuals. Behavioural states are defined *a priori* based on day of the year. Maximum movement distances under each behavioural state are defined for each individual based on a random sample from a Gaussian distribution. The model runs with a daily time step. Current location and day of simulation is noted, and the behavioural state defined. Environmental conditions (SST, food availability and depth) are sensed in the current and eight adjacent 1° grid cells. The probability of movement is sampled from a binomial distribution defined according to behavioural state and environmental conditions in the current location. The direction of movement is sampled based on discrete probabilities associated to each of the eight adjacent 1° cells, defined according to behavioural state and environmental conditions. Finally the distance moved is sampled from a Poisson distribution conditional on the maximum distance defined for each individual. The new location is then assigned from a linear vector originating at the current location with magnitude defined as the distance moved and direction as 45° increments reflecting the relative orientation of each of the eight potential adjacent cells.

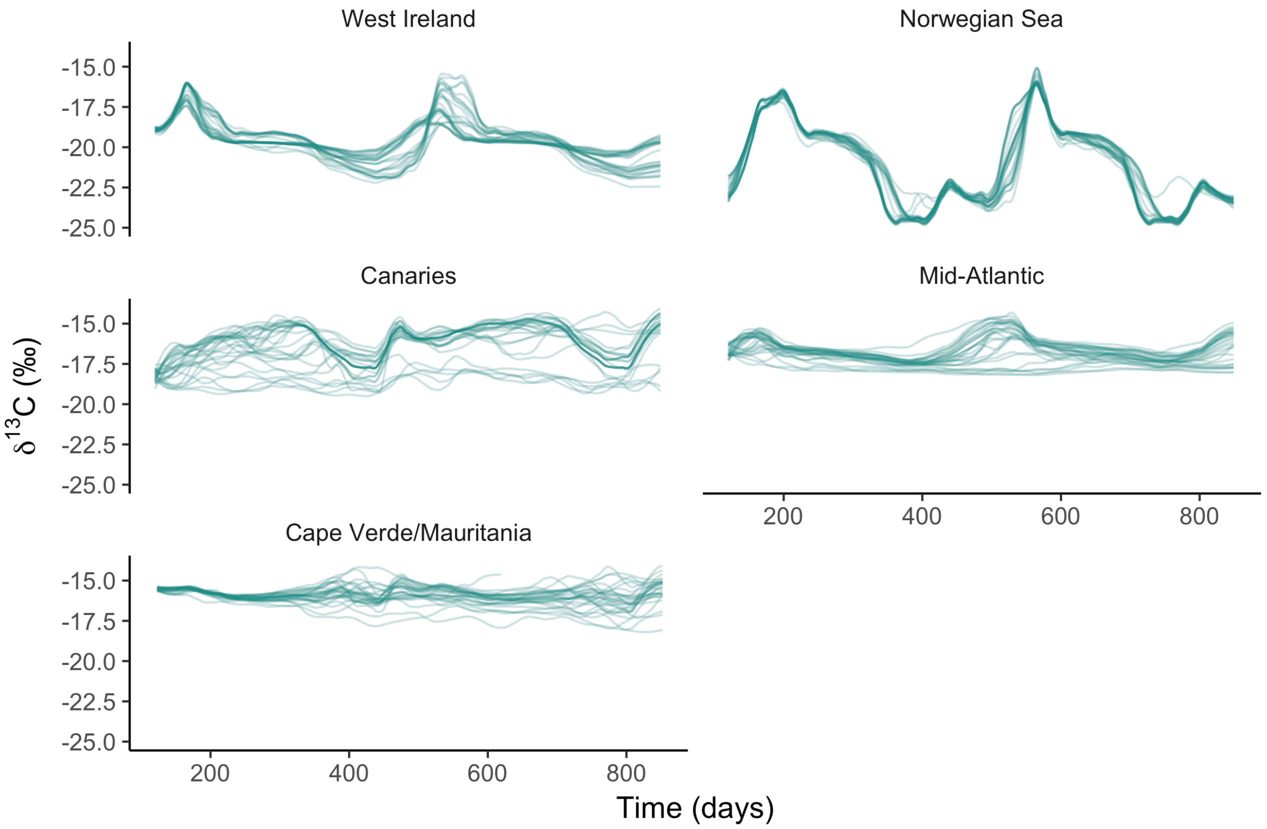


Figure S4: $\delta^{13}$C values expected in baleen plates for whales simulated with resident foraging models (i.e. restricting movements to broad regions) for west UK/Irish shelf (West Ireland), Norwegian/Barents Sea (Norwegian Sea), Canaries/west Azores (Canaries), Mid-Atlantic ridge (Mid-Atlantic), and the Cape Verde/Mauritanian upwelling area (Cape Verde/Mauritania), over a simulated two year period with daily resolution and 30 replicate simulations for each region. Simulated $\delta^{13}$C values are six month moving average values for the time series of simulated plankton $\delta^{13}$C values in that location.

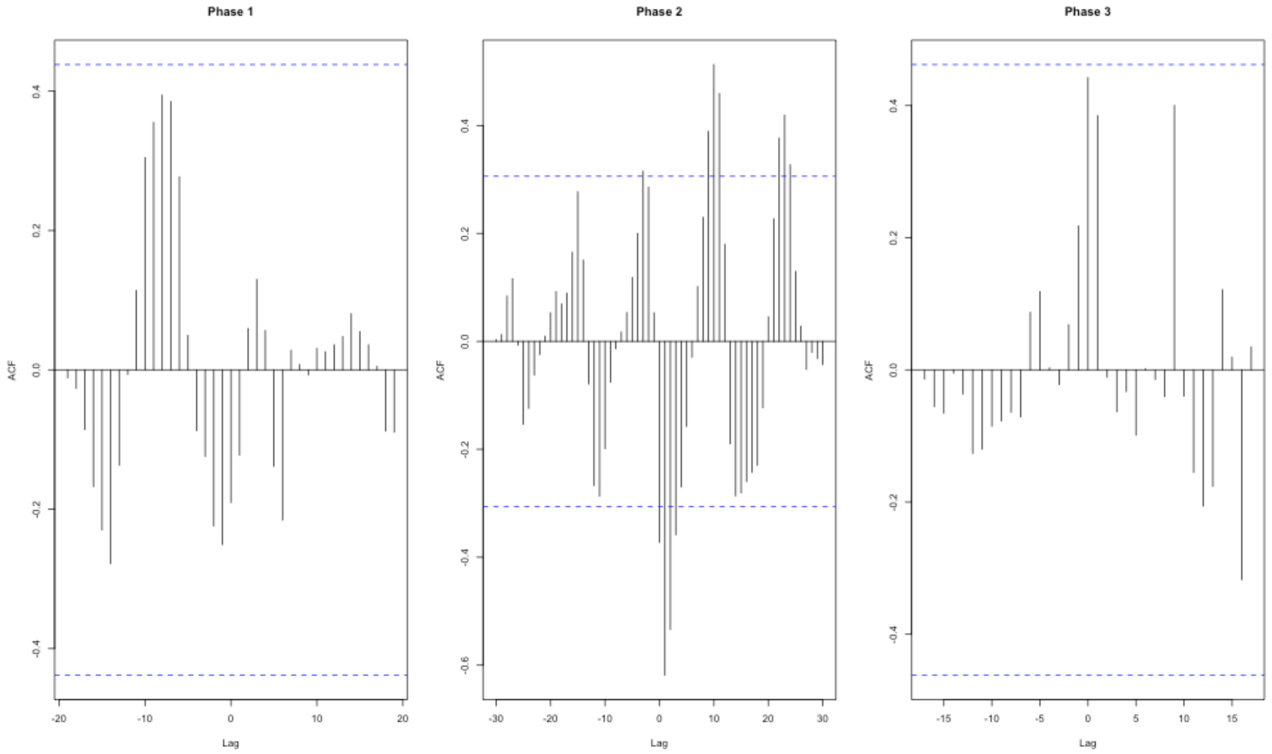


Figure S5: Cross correlation between $\delta^{13}$C and $\delta^{15}$N values for the three behavioural phases, showing limited covariation in behavioural phases one and three, but strong negative covariance in behavioural phase two with a periodicity of around 13cm.

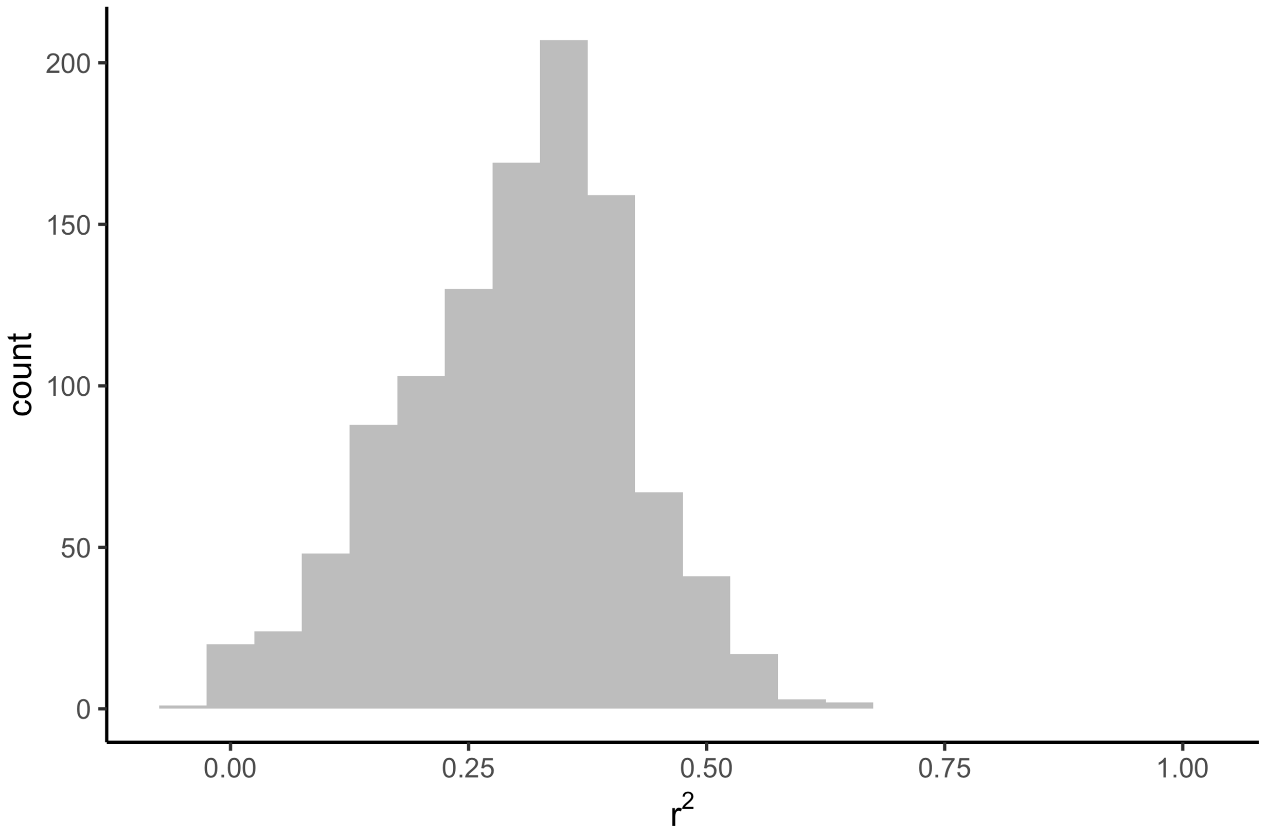


Figure S6: Histogram of $r^2$ values from linear regressions comparing model simulation $\delta^{13}C$ values to those of the real data (Figure 1).

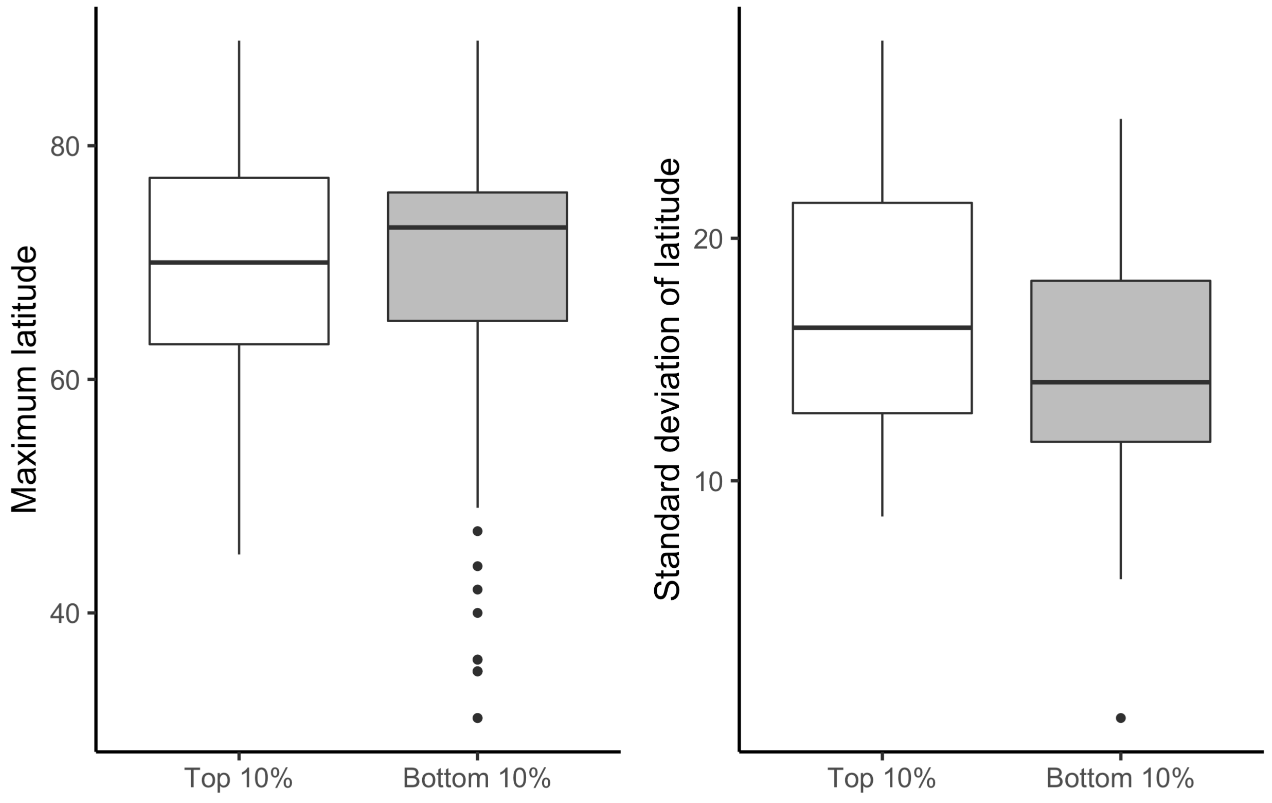


Figure S7: Boxplots showing the maximum and standard devation of latitudinal positions of simulated whales taken from the top 10%, and bottom 10%, of movement model simulations.

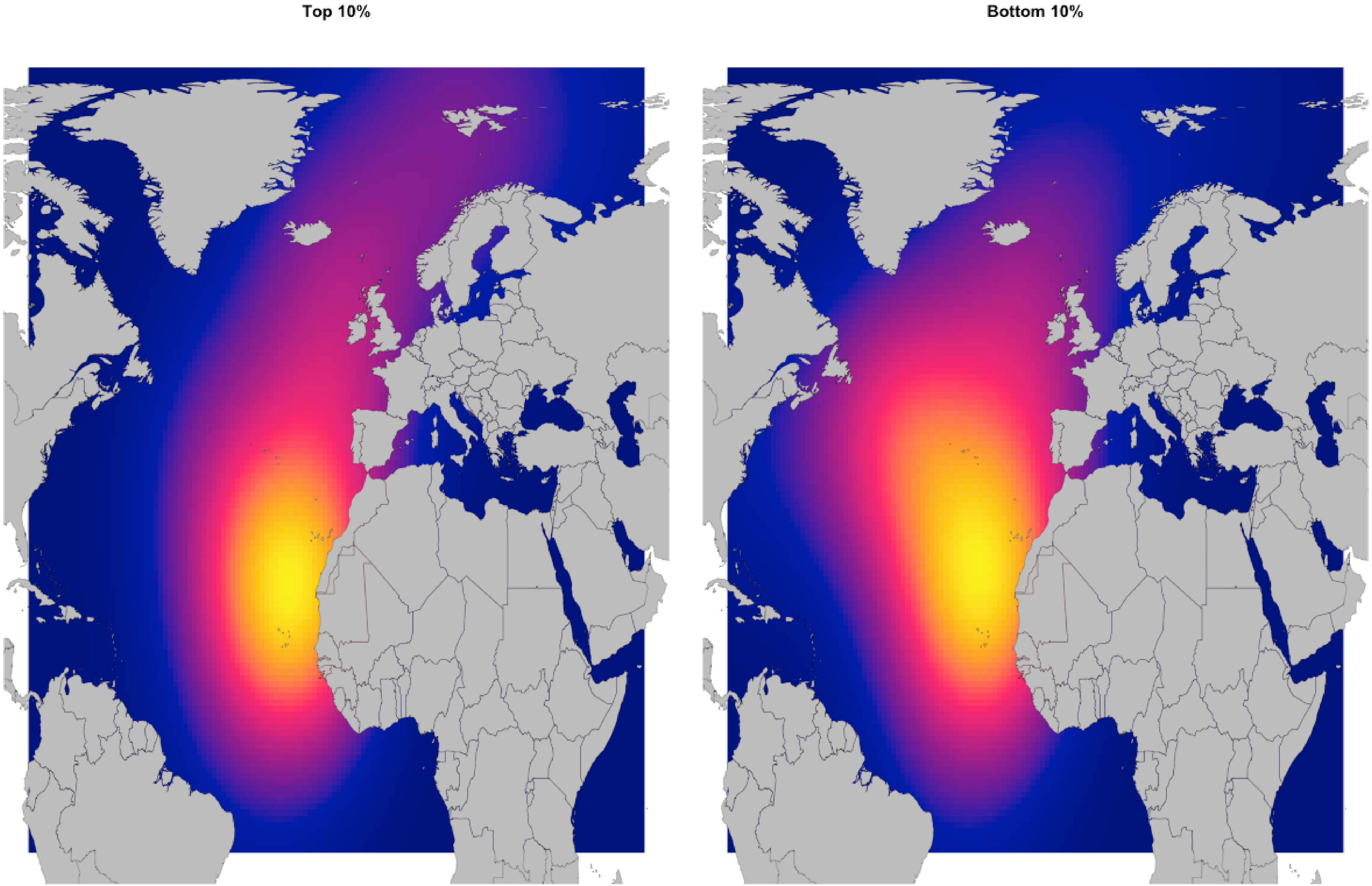


Figure S8: Kernal density plots showing the positions of simulated whales taken from the top 10%, and bottom 10%, of movement model simulations. High densities are in yellow, low densities are in blue.

# References

Magozzi, S., Yool, A., Vander Zanden, H., Wunder, M. & Trueman, C. 2017. Using ocean models to predict spatial and temporal variation in marine carbon isotopes. *Ecosphere*, **8**, e01763.
